# Potential Role of Glyphosate, Glyphosate-Based Herbicides, and AMPA in Breast Cancer Development: A Review of Human and Human Cell-Based Studies

**DOI:** 10.3390/ijerph21081087

**Published:** 2024-08-17

**Authors:** Hannah M. Schluter, Hajar Bariami, Hannah Lui Park

**Affiliations:** Department of Pathology and Laboratory Medicine, University of California, 839 Health Sciences Road, 218 Sprague Hall, Irvine, CA 92697, USA; hschlute@uci.edu (H.M.S.); hbariami@uci.edu (H.B.)

**Keywords:** glyphosate, aminomethylphosphonic acid (AMPA), glyphosate-based herbicides (GBHs), breast cancer risk, endocrine disruption, women’s health

## Abstract

The potential connection between exposure to glyphosate and glyphosate-based herbicides (GBHs) and breast cancer risk is a topic of research that is rapidly gaining the public’s attention due to the conflicting reports surrounding glyphosate’s potential carcinogenicity. In this review, we synthesize the current published biomedical literature works that have explored associations of glyphosate, its metabolite, aminomethylphosphonic acid (AMPA), and GBHs with breast cancer risk in humans and human cell-based models. Using PubMed as our search engine, we identified a total of 14 articles that were included in this review. In the four human studies, urinary glyphosate and/or AMPA were associated with breast cancer risk, endocrine disruption, oxidative stress biomarkers, and changes in DNA methylation patterns. Among most of the 10 human cell-based studies, glyphosate exhibited endocrine disruption, induced altered gene expression, increased DNA damage, and altered cell viability, while GBHs were more cytotoxic than glyphosate alone. In summary, numerous studies have shown glyphosate, AMPA, and GBHs to have potential carcinogenic, cytotoxic, or endocrine-disruptive properties. However, more human studies need to be conducted in order for more definitive and supported conclusions to be made on their potential effects on breast cancer risk.

## 1. Introduction

Glyphosate is the most widely used herbicide in the world; its global application in 2014 was enough to cover 22–30% of cultivated cropland [1]. Its use spans both agricultural and residential settings, including lawns and parks [2]. When glyphosate-tolerant crops were introduced in 1996, global glyphosate use in agriculture increased dramatically from 51 million kg in 1995 to 747 million kg in 2014 while non-agricultural use increased from 16 million kg in 1995 to 79 million kg in 2014 [1]. Glyphosate works by disrupting the shikimate pathway, which is used to biosynthesize aromatic amino acids in bacteria, plants, and certain fungi [3]. Throughout different nations, glyphosate, along with its metabolite, aminomethylphosphonic acid (AMPA), have been found in various foods, including grains, corn, soybean, and legumes [4,5,6,7,8]; thus, it is not surprising that human biomonitoring studies have found that they are frequently detected in urine [9,10,11,12,13,14].

There has been conflicting literature regarding the potential impact of glyphosate and AMPA on human health. A meta-analysis of human epidemiological studies by Zhang et al. (2019) [15] found that the highest cumulative exposure to glyphosate-based herbicides increased non-Hodgkin lymphoma risk by a statistically significant 41%, whereas another meta-analysis by Boffetta et al. [16] found no such relationship, claiming that the studies which have reported positive associations suffered from bias. A review by Mink et al. [17] did not find a consistent association between glyphosate and cancer risk. Other reviews have concluded that more information is needed to assess glyphosate’s safety and health risks [18,19]. However, a subset of these reports has disclosed possible conflict of interest due to researchers also serving as active or previous consultants for glyphosate producers [16,17]. Also, since the agricultural sector heavily relies on glyphosate, research into potential health implications may have economic consequences. The International Agency for Research on Cancer has deemed glyphosate as probably carcinogenic to humans (Group 2A) [20], but the United States Environmental Protection Agency (EPA) disagrees and has stated that glyphosate is unlikely to be carcinogenic [21]. However, the EPA’s February 2020 interim registration review decision on glyphosate was challenged and is now undergoing reconsideration after a June 2022 court decision [22]. 

Breast cancer is the most commonly diagnosed cancer in females in the world with an estimated 2.3 million new cases in 2020, comprising 11.7% of total new cancer cases [23]. In females, 1 in 4 cancer cases and 1 in 6 cancer deaths around the world are due to breast cancer, resulting in 685,000 deaths in 2020 [23]. With the majority of breast cancers believed to be due to non-genetic causes, there is much interest in investigating potential environmental/lifestyle risk factors [24,25,26,27]. Specific to breast cancer, there has only been one human study using biomonitoring to examine its potential relationship with glyphosate or AMPA, and it found that higher urinary AMPA levels were associated with greater breast cancer risk [28]. However, there have been a number of human studies examining the role of glyphosate and AMPA in physiological and cellular processes that can contribute to breast carcinogenesis, such as endocrine disruption, oxidative stress, and epigenetic changes, which will be discussed in this review. For example, recent studies have studied the potential effects of glyphosate and AMPA exposure during pregnancy, which can be a time of increased susceptibility to pesticide exposure [29]. Maternal urinary glyphosate and AMPA levels were associated with longer anogenital distances (AGD) in female newborns, suggesting endocrine disruption [9]. Urinary AMPA levels have also been found to be associated with urinary oxidative stress biomarkers [30], and oxidative stress is known to induce DNA damage that can contribute to carcinogenesis [31,32,33]. Our team’s study on postmenopausal women found that urinary glyphosate and AMPA were associated with DNA methylation levels in the promoters of several genes associated with cancer and endocrine disruption [11]. In addition, many human cell-based studies have examined the effect of glyphosate or AMPA on endocrine disruption, oxidative stress, and other cellular processes related to carcinogenesis, with some studies finding an effect while others did not. These human and human cell-based studies are the subject of this review.

Considering glyphosate’s widespread use, the controversy regarding its carcinogenic status, and breast cancer’s significant prevalence, the purpose of this review was to synthesize the current literature examining the relationships between glyphosate, AMPA, and/or glyphosate-based herbicides and breast cancer risk or processes associated with mammary carcinogenesis.

## 2. Materials and Methods

Due to the time-sensitive manner of our chosen subject, we sought to provide a comprehensive and descriptive overview of the existing literature. The updated PRISMA 2020 guidelines [34] were used to address the majority of the aspects seen in a systematic review; however, biases and certainty were not assessed.

### 2.1. Inclusion/Exclusion Criteria

Peer-reviewed studies in which glyphosate, glyphosate-based herbicides (GBHs), or AMPA levels were measured in relation to female breast cancer risk and/or examined for their effects on related physiological or molecular processes in humans or human cell culture models were included in this review. The types of studies included cross-sectional, cohort, case–control, and experimental studies. All studies had to (i) report a relative risk or risk ratio (RR), hazard ratio (HR), or odds ratio (OR) with 95% confidence intervals on the association between glyphosate/GBH/AMPA and health outcome in humans; or (ii) examine potential relationships between glyphosate/GBH/AMPA and cellular processes involved in carcinogenesis in humans or using human cell culture models. We excluded articles if they (i) were a review; (ii) only used cell models derived from plant and/or animal cells; (iii) only used non-breast human cell lines; or (iv) were not on a relevant topic. 

### 2.2. Search Strategy 

The following search terms were entered into the advanced search function in PubMed: ((Glyphosate) OR (Aminomethylphosphonic acid)) AND ((Women) OR (Human)) AND ((Breast) OR (Mammary) OR (Endocrine)) on 25 July 2022 to include all available articles up to and including this date. These search terms were used to identify relevant papers that focused on human or human cell-based research and breast cancer, mammary carcinogenesis, or endocrine disruption. Two researchers (HMS, HB) independently screened through this list according to the inclusion/exclusion criteria described above to identify the set of studies that would be included in this review.

### 2.3. Data Extraction

Two researchers (HMS, HB) independently extracted information from each of the articles that were included in the review. Any discrepancies were resolved through discussion among HMS, HB, and an additional team member (HLP). When there was uncertainty regarding information in any of the studies reviewed and a confident consensus between HMS and HB could not be made, HLP was consulted. For each of the articles, information that was extracted included: first author/year published, type of chemical (glyphosate, AMPA, and/or GBH) used, and outcome measures/results. For the human studies, the following data were specifically extracted: study design, sample size, setting, population, time period, exposure assessment and category, strengths, and weaknesses. For the human cell studies, the following additional data were extracted: cell line(s), treatment(s), assay(s)/technique(s) used, and proposed cellular mechanisms. 

### 2.4. Outcome Measures

The outcome measures for the human studies included measures of breast cancer risk, endocrine disruption, oxidative stress biomarkers, and DNA methylation. Specific outcomes seen across multiple human cell line studies included cell viability, aromatase activity, estrogenic activity, genotoxicity, and gene expression.

## 3. Results

**Figure 1:**
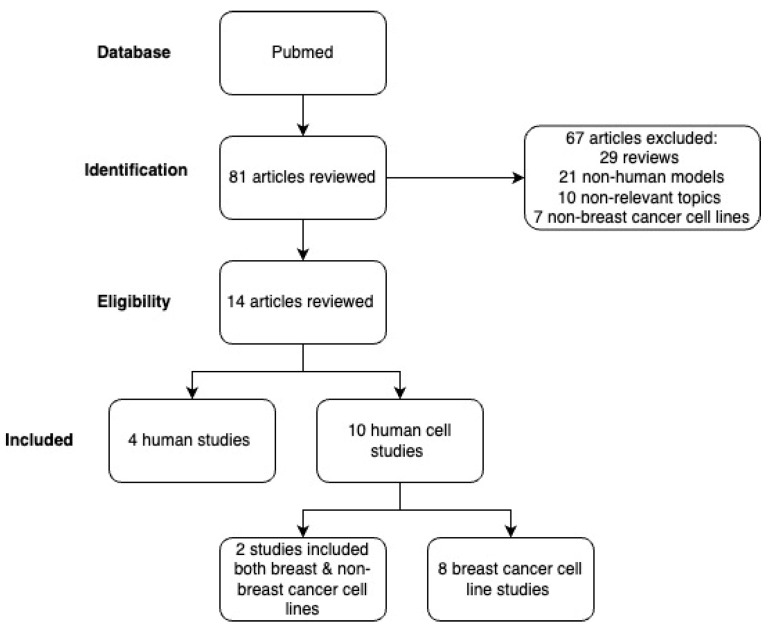
PRISMA (2020) Flow Diagram for Selection Process.

Our search resulted in a total of 81 articles, and through the screening process, 14 studies (4 human and 10 human breast cell-based) were included in this review (Figure 1).

### 3.1. Human Studies

Each of the four human studies assessed exposure to glyphosate and/or AMPA by measuring their concentrations in participants’ urine. The study locations ranged from Southern California [11] to Hawaii [28], Puerto Rico [30], and across multiple sites in the United States [9]. A comprehensive summary of the human studies is shown in Table 1. 

Franke et al. conducted a nested case–control study using urine samples from predominantly postmenopausal women living in Hawaii, who participated in the biospecimen subcohort of the Multiethnic Cohort (MEC), to investigate the potential association between urinary AMPA concentrations and breast cancer risk [28]. Based on 124 breast cancer cases and 126 matched controls, they found that the geometric mean urinary AMPA concentration following creatinine adjustment was 40% higher in cases than controls, but this was not statistically significant (*p* = 0.21). However, participants in the first and second highest quintile of urinary AMPA levels had a statistically significant higher breast cancer risk compared to those in the lowest quintile (OR 4.49 [95% CI 1.46–13.77] and OR 3.03 [95% CI 1.02–9.03], respectively). While these findings suggest that AMPA exposure may be associated with increased breast cancer risk, the investigators pointed out that their results require confirmation in a larger population to increase study power.

**Table 1:** Summary of human studies on glyphosate/AMPA exposure and outcomes related to breast cancer risk/endocrine disruption.

First Author, Year Published	Study Design, Setting, Population, Period	Exposure Assessment	Glyphosate, AMPA, and/or GBH(s)	Method (LOD/LOQ), Corrections for Urine Concentration	Exposure Range/Median	Outcome Measures	Results	Strengths	Weaknesses
Eaton et al., 2022 [30]	Prospective cohort study; Northern Karst region of Puerto Rico; 205 pregnant women (18–40 years old) from the PROTECT birth cohort; 2012–2017	Urinary Gly and AMPA levels at 16–20 (Visit 1) and 24–28 (Visit 3) weeks gestation	Gly and AMPA	GC-MS/MS (LOD: 0:20 µg/L), corrected for specific gravity	Median Gly level was 0.50 ng/mL (IQR: 0.29, 0.79); median AMPA level was 0.26 ng/mL (IQR: 0.17, 0.49).	Levels of oxidative stress biomarkers	An IQR increase in urinary AMPA was associated with higher 8-iso-PGF2α metabolite levels in mixed-effects model (6.71% [95% CI 1.51–12.17%]) and at visit 3 specifically (9.03% [95% CI 0.55–18.21%]). An IQR increase in Gly was associated with higher 8-iso-PGF2α levels only at visit 3 (7.23% [95% CI 0.11–14.86%]).	One of few studies to measure human exposure to both Gly and AMPA. First human study to look at association between AMPA and oxidative stress biomarkers. The use of two time points allowed for greater statistical power and determination of susceptibility periods.	Observational study so causality is indeterminable. The 8-iso-PGF2α/PGF2α ratio method used to show differences between oxidative stress and inflammation may not be an accurate representation.
Franke et al., 2021 [28]	Nested case–control pilot study; Hawaii; 250 women (45–75 years old) from the Multiethnic Cohort; 2001–2006	Overnight urinary AMPA concentrations	AMPA	LC/HRAM-MS (LLOQ: 0.001 ng/mL), uncorrected and corrected for urinary creatinine	Urinary AMPA levels ranged from <LLOQ to 3698 ng/L. Adjusted geometric mean in cases was 0.087 ng/mg (95% CI 0.055–0.119); in controls was 0.063 ng/mg (95% CI 0.032–0.095).	Incident breast cancer	Urinary AMPA was associated with increased breast cancer risk. Highest vs. lowest quintile—OR 4.49; 95% CI 1.46–13.77 Second vs. lowest quintile—OR 3.03; 95% CI 1.02–9.03	First prospective study to look at associations between urinary AMPA levels and breast cancer risk (measured by liquid chromatography mass spectrometry).	Observational study so causality is indeterminable. Used only one urine sample, may not be representative of usual AMPA exposure.
Lesseur et al., 2021 [9]	Pilot study nested within prospective cohort; UCSF, URMC, UMN, UW; 94 pregnant women and their term infants from The Infant Development and the Environment Study (TIDES) pregnancy cohort; August 2010–August 2012	Urinary Gly and AMPA levels in 2nd trimester mothers	Gly and AMPA	UPLC-MS/MS (Gly LOD: 0.014 ng/mL; LOQ: 0.041 ng/mL), (AMPA LOD: 0.013 ng/mL; LOQ: 0.04 ng/mL), corrected for specific gravity	Urinary Gly levels ranged from 0.01 to 1.9 ng/mL (median: 0.22 ng/mL); AMPA levels ranged from 0.01 to 6 ng/mL (median: 0.14 ng/mL).	Anogenital distance (AGD) in newborns	Maternal urinary Gly was associated with longer (unadjusted) AGD-AC (*p* = 0.05). Maternal urinary AMPA was associated with longer AGD-AF (*p* = 0.01).	First study to look at association between maternal urinary Gly/AMPA levels and AGD in human newborns. Multicenter TIDES study included data from various geographic areas of the US.	Observational study so causality is indeterminable. Only one urine sample in 2nd trimester, which does not correspond with masculinization programming window.
Lucia et al., 2022 [11]	Cross-sectional study; Southern California; 392 postmenopausal women aged 45 to 66 years; 2017–2019	First morning urinary Gly and AMPA levels on 2 days within a 10-day period	Gly and AMPA	LC-MS/MS (Gly—LOD: 0.014 ng/mL; LOQ: 0.041 ng/mL), (AMPA—LOD: 0.013 ng/mL; LOQ: 0.040 ng/mL), adjusted for urinary creatinine	Median Gly level was 0.12 ng/mL (IQR: 0.06, 0.22) and median AMPA level was 0.06 ng/mL (IQR: 0.02, 0.12).	DNA methylation level at >850,000 CpG sites	Urinary AMPA was associated with increased epigenetic age acceleration (*p* = 0.04). Urinary Gly was associated with DNA methylation of regions in the promoters of *MSH4*, *KCNA6*, *ABAT*, and *NDUFAF2/ERCC8*. Urinary AMPA was associated with DNA methylation in the *ESR1* promoter.	Largest study on urinary Gly and AMPA levels in a nonagricultural setting. Most recent practices were used to analyze DNA methylation. Two urine samples were used per participant to provide a better representation of usual Gly/AMPA exposure.	Observational study so causality is indeterminable. Also indeterminable if differences in DNA methylation will affect gene expression due to lack of gene expression data.

Lesseur et al. conducted a nested cohort study that looked at second trimester maternal urinary glyphosate and AMPA levels in 94 randomly selected participants from The Infant Development and the Environment Study (TIDES) pregnancy cohort [9]. Among the female infants (*n* = 45), high maternal urinary AMPA levels were associated with longer anogenital distance from the anus to the posterior fourchette (AGD-AF) in unadjusted and adjusted models (*p* = 0.01 for both). High maternal urinary glyphosate level was also associated with longer anogenital distance from the anus to the anterior clitoral surface (AGD-AC) (*p* = 0.05), but this was not statistically significant (*p* = 0.07) when adjusted for infant age and size at AGD exam. Among the male infants (*n* = 49), glyphosate and AMPA were not associated with differences in AGD. In both sexes, longer AGD reflects greater androgen exposure, and studies have shown that AGD can serve as a marker for in utero exposure to endocrine-disrupting chemicals [35,36]. Thus, the association Lesseur et al. [9] found between longer female infant AGD-AF and maternal AMPA exposure could indicate AMPA’s potential endocrine-disruptive properties. However, no studies to date have shown a direct link between infant AGD and breast cancer risk.

Eaton et al. examined the association between urinary glyphosate and AMPA levels with oxidative stress biomarkers in pregnant women [30]. Urine samples were analyzed from 205 pregnant women from the Northern Karst region of Puerto Rico participating in the PROTECT birth cohort study at 16–20 and 24–28 weeks gestation. At 24–28 weeks gestation, an interquartile range (IQR) increase of glyphosate was associated with a 7.23% higher 8-iso-PGF2α concentration (95% CI 0.11–14.86%) and a 10.90% (95% CI 0.25–22.69%) increase in absolute chemical lipid peroxidation. An IQR increase in AMPA was found to be significantly associated with a 6.71% (95% CI 1.51–12.17%) higher concentration of the main 8-iso-PGF2α metabolite, 2,3-dinor-5,6-dihydro-15-F_2t_-isoprostane. In addition, the second and third tertiles of AMPA were associated with significant increases in the concentration of 2,3-dinor-5,6-dihydro-15-F_2t_-isoprostane compared to the lowest exposure tertile (12.85% [95% CI 0.63–26.55%]) and (15.20% [95% CI 1.83–30.32%]), respectively. Prolonged oxidative stress causes DNA mutations and cell damage, which can result in chronic inflammation and increase cancer risk [31,32,33]. Therefore, the associations between oxidative stress biomarkers and glyphosate/AMPA exposure observed by Eaton et al. warrant further investigation.

Lucia et al. conducted a cross-sectional study that examined the relationship between glyphosate and AMPA exposure and blood DNA methylation patterns in 392 postmenopausal women in Southern California [11]. They found that urinary glyphosate level was associated with the methylation level of 24 CpG sites in the promoters of genes including some related to cancer, such as *SF3B2*, *MSH4*, and *ERCC8*. Urinary AMPA level was associated with the methylation level of a region of the *estrogen receptor 1* (*ESR1*) promoter. AMPA was also associated with increased epigenetic age acceleration (*p* = 0.04), which has been shown to be associated with cancer risk [37,38], but glyphosate was not. Since epigenetic mechanisms can contribute to cancer development [39], studying DNA methylation patterns associated with glyphosate/AMPA exposure can help elucidate their potential health effects. While tissue-specific methylation (in breast tissue) would provide more direct insights, peripheral blood methylation status could reflect systemic epigenetic alterations associated with cancer susceptibility. In addition, the AMPA-associated hypomethylation at the *ESR1* promoter is interesting, given the potential link between AMPA and breast cancer risk [28] and the potential for glyphosate/AMPA-induced endocrine disruption, reviewed in this paper.

### 3.2. Human Breast Cell Studies

Our search resulted in 10 human cell studies that used human breast cancer or immortalized breast cells. In each of the studies, cells were treated with glyphosate, GBHs (e.g., Roundup, Wipeout), and/or AMPA. Outcome measures included: cell viability/proliferation, apoptosis, gene expression, and aromatase activity. The results for the human cell studies are summarized in Table 2.

Six of them measured cell viability (including cell proliferation and cytotoxicity). Stur et al. found that Roundup (a GBH) and AMPA reduced cell viability and caused cellular damage in MDA-MB-468, a hormone-independent breast cancer cell line, and MCF-7, a hormone-dependent breast cancer cell line, and Roundup was more toxic than AMPA [40]. Similarly, Coppola et al. observed a reduction in cell viability in MCF-7 and MCF-12A (non-tumorigenic) cells upon glyphosate treatment [41]. In contrast, Mesnage et al. (2017) [42] and Thongprakaisang et al. [43] found that glyphosate induced cell proliferation in hormone-dependent breast cancer cell lines (MCF-7, T47D) but not in hormone-independent ones (MDA-MB-231). Furthermore, Lin and Garry observed that glyphosate and Roundup induced cell proliferation at relatively low concentrations and reduced cell viability at higher concentrations through inducing necrosis in MCF-7 cells [44]. Although De Almeida et al. found that glyphosate and GBHs did not significantly affect cell viability, they reported that these compounds induced DNA damage in MDA-MB-231 but not MCF-7 cells [45]. Looking at other measures of cytotoxicity, Coppola et al. found that glyphosate reduced intracellular ATP levels in MCF-7 cells and increased apoptosis but reduced reactive oxygen species (ROS) production in MCF-12A cells [41]. Overall, there was not a consensus among the six studies regarding cell viability even when the same cell lines were used: two [40,41] reported a decrease for hormone-independent and/or hormone-dependent lines, two [42,43] found an increase for hormone-dependent but not hormone-independent lines, one [44] reported an increase at low concentrations and a decrease at high concentrations for a hormone-dependent line, and one [45] found no change.

Four studies measured estrogenic activity in immortalized breast or breast cancer cell lines. Zhang et al. (2020) reported that glyphosate did not increase estrogen-responsive transcription nor hinder binding between 17β-estradiol (E2) and estrogen receptor α (ERα) in hormone-dependent MELN cells, but it inhibited aromatase activity [46]. In contrast, Mesnage et al. (2017) found that glyphosate, but not Roundup, increased estrogen response element (ERE)-mediated expression in hormone-dependent T47D-KBluc cells and altered gene expression in MCF-7 cells but not through ERα activation [42]. Coppola et al. observed that glyphosate increased E2 secretion and altered nuclear receptor [ERα, ERβ, androgen receptor (AR), aryl hydrocarbon receptor (AhR), and progesterone receptor (PgR)] gene expression in MCF-7 and MCF-12A cells [41]. Similarly, Thongprakaisang et al. reported that glyphosate induced ERE transcription activity in T47D-KBluc cells and increased ERα and ERβ protein levels in T47D cells [43]. Examining anti-androgenic effects on AR, Gasnier et al. reported that glyphosate and Roundup reduced dihydrotestosterone (DHT)-mediated transcriptional activity in androgen-responsive MDA-MB453-kb2 cells [47]. Taken together, glyphosate seems to have endocrine-disruptive properties, but the effects and mechanisms are unclear, as two studies [42,43] found that glyphosate induced estrogen-responsive transcription, whereas one study [46] found no effect on transcription but reported reduced aromatase activity.

Two articles measured expression of other genes. Hokanson et al. reported that *HIF1* was upregulated and *CXCL12* and *EGR1* were downregulated upon treatment with glyphosate in MCF-7 cells [48]. Tumors, including breast cancer, are often hypoxic compared to healthy tissue [49]. In hypoxic cancer cells, HIF1 (hypoxia-inducible factor 1) regulates genes associated with metabolic reprogramming that promote tumor progression through proliferation and angiogenesis [50]. However, CXCL12 (stromal cell-derived factor 1) promotes tumor cell survival by inhibiting apoptosis, so *CXCL12* downregulation would not support tumor progression [51]. In certain cases, EGR1 (early growth response factor 1) has been shown to induce tumor cell apoptosis, whereas it promotes tumor proliferation and angiogenesis in hypoxic conditions [52]. Thus, additional studies need to be conducted to clarify the interaction of gene expression changes associated with glyphosate. Stur et al. found that Roundup deregulated 11 canonical pathways (NOTCH, WNT, Hedgehog, TGF-β, MAPK, JAK-STAT, PI3K-AKT, RAS, cell cycle, apoptosis, and DNA damage control) in MCF-7 and MDA-MB-468 cells, whereas AMPA exposure resulted in less differentially expressed genes, most of which were associated with metabolism in MDA-MB-468 cells [40]. These combined findings point to glyphosate and AMPA inducing genes involved in cell growth and metabolic changes in breast cancer cells, but more research is needed to determine how this could affect tumor initiation and/or progression. One article examined the assertion that glyphosate substitutes for glycine in polypeptide chains leading to protein misfolding and toxicity; the authors conducted a proteomics analysis of MDA-MB-231 cells and determined that glyphosate did not substitute for glycine [53].

**Table 2:** Summary of human cell studies on glyphosate/GBH/AMPA exposure and outcomes related to mammary carcinogenesis.

First Author, Year Published	Chemical Used	Cell Line(s)	Treatment	Outcome Measures	Assays/Techniques Used	Main Findings	Proposed Mechanisms
Antoniou et al., 2019 [53]	Gly	MDA-MB-231	100 mg/L Gly for 6 days	Global proteome changes; presence of glyoxylate-modified cysteines or glycine to Gly substitutions	RPLC-MS/MS/MS	No global proteome changes; Gly did not substitute for glycine nor did glyoxylation occur in proteins.	Potential Gly toxicity is not through glycine substitution.
Coppola et al., 2022 [41]	Gly	MCF-7 and MCF-12A	For cell viability, apoptosis/necrosis, and ATP levels: 230 pM, 2.3 nM, 23 nM, 230 nM, or 2.3 µM of Gly For other outcomes: 2.3, 23, or 230 nM of Gly	Cell viability; cell proliferation; apoptosis and necrosis; ATP levels; intracellular ROS levels; estradiol (E2) secretion; gene expression of nuclear receptors (ERα, ERβ, AR, AhR and PgR)	MTS; CyQuant; RealTime-Glo Annexin V Apoptosis and Necrosis; Mitochondrial ToxGlo; ROS Detection; Estradiol ELISA; real-time PCR	MCF-7: Gly reduced cell viability at 2.3 nM, 230 nM, and 2.3 µM and decreased cell proliferation at 230 pM; 230 nM and 2.3 µM decreased intracellular ATP levels; 2.3 nM increased E2 secretion; 2.3 nM downregulated ERα and Erβ, whereas 23 nM upregulated ERα; 23 and 230 nM upregulated AR; 2.3 and 230 nM downregulated PgR; 23 nM upregulated AhR MCF-12A: Gly reduced cell proliferation at 23 nM, 230 nM, and 2.3 µM; 2.3 nM increased apoptosis at 7 and 8 h; all concentrations reduced ROS; 230 nM increased E2 secretion; all concentrations (2.3, 23, and 230 nM) upregulated ERα and ERβ and downregulated PgR; 23 and 230 nM downregulated AR; 2.3 and 23 nM downregulated AhR	Gly may increase mitochondrial membrane permeability, increasing intracellular calcium concentrations and thus reducing ATP synthesis. Gly might alter balance of ERα and ERβ receptors, affecting mammary gland development. Gly increased E2 secretion, which could activate vascular endothelial growth factor transcription, and may lead to mammary gland angiogenesis.
De Almeida et al., 2018 [45]	Gly, Roundup, and Wipeout	MCF7 and MDA-MB-231	0–500 µg/mL of Gly, Roundup (Ro), or Wipeout (Wo) for 24 h	Cell viability and genotoxicity	MTT; comet	Gly (500, 1000 µg/mL), Ro (500, 800 µg/mL), and Wo (500 µg/mL) induced DNA damage in MDA-MB-231. No cytotoxic effects were observed for MCF7 and MDA-MB-231.	Gly, Ro, and Wo might cause toxic effects through non-estrogenic mechanisms.
Gasnier et al., 2009 [47]	Gly and 4 Roundup (R) formulations	MDA-MB453-kb2	2% solutions (and consecutive dilutions up to 10^−7^) of 1 of 4 R formulations (7.2 g/L, 360 g/L, 400 g/L, 450 g/L of Gly) or Gly alone (360 g/L) for 24 h	Anti-androgenic effects (on androgen receptor)	Luciferase reporter gene	All R formulations and Gly lowered DHT-mediated transcriptional activity.	Gly might bind to a steroid receptor. Adjuvants might form vesicles that intensify Gly effects by enhancing stability, cell penetration, and bioavailability.
Hokanson et al., 2007 [48]	Gly	MCF-7	Gly at 0.1, 0.01, 0.001 or 0.0001% dilutions of the 15% stock for 18 h with or without 3 × 10^−10^ M E2	Gene expression	DNA microarray; quantitative real-time PCR	At 0.00023% Gly, HIF1 was upregulated (more than twofold) and CXCL12 and EGR1 downregulated (more than 50%). Gly plus estrogen had greater effects than estrogen alone.	Gly and estrogen might synergistically affect gene expression, potentially damaging adult and fetal cells. Altered levels of EGR1, HIF1, and CXCL12 may initiate apoptosis, increase tumor angiogenesis, inactivate tumor suppressor genes, and disrupt immune surveillance.
Lin and Garry, 2000 [44]	Gly and Roundup	MCF-7	Different concentrations that included up to 10 µg/mL for 72 h (cell viability) or 7 days (cell proliferation)	Cell viability; cell proliferation; apoptosis	FACS	Gly (0.228–2.28 µg/mL) and Roundup (1–10 µg/mL) induced cell proliferation in CD-treated (lacking estrogen) and non-CD-treated cultures. At cytotoxic concentrations, they induced necrosis (reduced cell viability and loss of cell membrane integrity).	Gly and Roundup might use a non-estrogenic mechanism to induce cell proliferation. Dependence on estrogenic mechanism is unknown for cytotoxic effects.
Mesnage et al., 2017 [42]	Gly, 4 GBH formulations, POEA (adjuvant)	MCF-7, T47D, T47D-KBluc, and MDA-MB-231	10^−6^ to 10^7^ μg/L of Gly, GBH, or adjuvants for 6 days (ER-mediated cell proliferation), 24 h (ERE-mediated transcription), or 48 h (transcriptomics analysis)	ER-mediated cell proliferation (MCF-7, T47D, MDA-MB-231); transcriptomics analysis (MCF-7); ERE-mediated transcription (T47D-KBluc); molecular dynamic simulations	E-screen-MTT; ERE-luciferase reporter gene; microarray; RNA sequencing	Gly ≥ 10,000 μg/L promoted proliferation of MCF-7 cells and T47D less so; no effect in MDA-MB-231. Gly, but not Roundup nor POEA, increased ERE-mediated expression at ≥1000 μg/L; ER antagonist ICI 182,780 blocked this. Gly altered MCF-7 gene expression but not through ERα activation. Gly binding energy calculation predicts a weak and unstable interaction with ERα‘s active site.	Gly might activate ERα via a ligand-independent mechanism, possibly through the PKA pathway, but only at relatively high concentrations so humans exposed to Gly at typical levels would not be expected to exhibit ER activation.
Stur et al., 2019 [40]	AMPA and Roundup	MDA-MB-468 and MCF-7	0.01 to 10 mM of AMPA or 0.01% to 0.3% of Roundup for 3, 15, 24, and 48 h	Cell viability; gene expression	MTT; microarray	Roundup was more toxic than AMPA. After 48 h of 0.05% Roundup (1.1 mM Gly) exposure, 11 canonical pathways * were deregulated in both cell lines, including a more pronounced downregulation of cyclins and DNA damage repair pathways in MCF-7. 48 h of 10 mM AMPA exposure resulted in less differentially expressed genes, with most associated with metabolism in MDA-MB-468.	Roundup may deregulate ER-independent pathways related to cell cycle, DNA repair, and metabolism, which could change mitochondrial oxygen consumption, cause hypoxia, increase ROS, prevent DNA repair resulting in mutation buildup, and induce cell death.
Thongprakaisang et al., 2013 [43]	Gly	T47D, T47D-KBluc, and MDA-MB-231	10^−12^ to 10^−6^ M of Gly or estradiol (positive control)	Cell viability/number; estrogenicity and anti-estrogenicity of Gly (estrogen response element (ERE) transcription activity); ERα and ERβ expression	MTT; ERE-luciferase reporter gene; Western blot	Without E2 in the medium, Gly resulted in a 15–30% proliferation of T47D cells. Gly had no effect on the growth of hormone-independent MDA-MB-231 cells both in the absence/presence of E2. 1 nM of ER antagonist ICI 182780 weakened E2’s and Gly’s proliferative effects, while 10 nM completely inhibited the latter. In T47D-KBluc cells, Gly induced ERE activation 5–13-fold. When co-incubated, Gly suppressed E2-induced ERE activation. In T47D cells, Gly increased ERα and ERβ levels in a dose-dependent manner after 6 h exposure, but only ERα levels increased at the highest Gly concentration (10^−7^ M) after 24 h exposure.	Gly’s proliferative and stimulatory effects may occur via ER signaling since an ER antagonist inhibited this proliferation. At the ligand site of ERs, hydrophilic Gly may bind in a polar pocket. When the endogenous agonist E2 is present, Gly acts as an antagonist. Gly acts like a weak xenoestrogen that quickly activated ERβ while ERα activation was slower and longer.
Zhang et al., 2020 [46]	Gly	MELN	500, 1000, and 1500 nM Gly	Aromatase activity; estrogenic activity; molecular dynamics	ELISA; luciferase reporter gene	Gly inhibited up to 30% of aromatase activity in a dose-dependent manner but did not interfere in the binding between E2 and ERα nor increase estrogen-responsive transcription.	Gly inhibits aromatase by potentially binding to an allosteric site.

* Details are mentioned in the text.

## 4. Discussion

As the first comprehensive and detailed compilation of human and human cell-based studies that focus on the potential relationships between glyphosate, GBHs, and AMPA and breast cancer risk or development, this review highlights the importance of further studies to determine the potential roles of these compounds in breast cancer etiology. While the number of human studies that qualified to be reviewed was limited and their designs varied, each study used urinary measurements of glyphosate and/or AMPA as their form of exposure assessment. Eaton et al. [30] and Lesseur et al. [9] utilized pregnant women in their studies, while Franke et al. [28] and Lucia et al. [11] used (mostly) postmenopausal women between the ages of 45–75 years and 45–66 years, respectively. Collectively, higher urinary levels of glyphosate and/or AMPA were associated with breast cancer risk [28], higher levels of oxidative stress biomarkers [30], endocrine disruption [9], and DNA methylation differences [11]. The study by Franke et al. [28] was the only study that examined the potential relationship between glyphosate/AMPA exposure and breast cancer risk, and their results showing that breast cancer risk was 4.5-fold higher in women with the highest vs. lowest quintile for urinary AMPA warrant replication in a separate cohort. Lucia et al. [11] found that urinary glyphosate was associated with the methylation level of 24 CpG sites in the promoters of multiple genes including *MSH4*, which is associated with cancer. Urinary AMPA was associated with hypomethylation in the *ESR1* promoter [11], which has been linked to breast cancer risk [54,55] and endocrine disruption [56]. The studies by Eaton et al. [30] and Lesseur et al. [9] on pregnant women portrayed the potential downstream effects glyphosate exposure could have on women and infants.

In the course of searching for human breast cell studies that met our inclusion criteria, we found that two of the studies we included in this review also included the examination of non-breast human cell studies, and there were seven other human cell studies that met our inclusion criteria except that they were on non-breast human cells. These other papers contained interesting results as well. All of these studies measured cell viability, and most reported that GBHs and glyphosate reduced cell viability, with Roundup and other GBHs being more toxic than pure glyphosate [47,57,58,59,60,61]. For example, Defarge et al. (2018) found that HEK293 cells were killed by GBHs within 90 min but not by glyphosate alone [62]. However, De Almeida et al. observed a dose-independent increase in HEC1A cell viability upon exposure to Wipeout (a GBH) along with a dose-independent reduction in HEC1A cell viability upon glyphosate treatment, whereas Roundup showed no significant effect [45]. In addition, Sritana et al. reported that glyphosate induced cell proliferation at lower concentrations, whereas higher concentrations reduced cell viability in HuCCA-1 cells [63]. One study reported that average Caco2 cell size decreased for all glyphosate and Roundup doses [61]. 

Five studies reported on aromatase activity [47,57,59,60,62], and three found that it significantly decreased upon treatment with GBHs and glyphosate, and GBHs had a stronger effect than glyphosate alone [57,60,62]. Furthermore, Richard et al. found that Roundup, but not glyphosate, reduced aromatase activity and mRNA levels in JEG3 cells, whereas both decreased aromatase activity in human placental microsomes, with Roundup having a greater inhibitory effect [59]. Similarly, Gasnier et al. observed that in HepG2 cells, Roundup lowered aromatase activity and estradiol-mediated transcriptional activity, indicating anti-estrogenic effects on ERα and ERβ, while glyphosate did not [47].

Other cancer-related cellular processes were also examined. For example, genotoxicity was measured in two studies. Gasnier et al. showed that R400 (a highly potent Roundup formulation) increased the amount and severity of DNA damage in HepG2 cells [47], while De Almeida et al. showed that glyphosate and GBHs induced DNA damage in HEC1A cells [45]. Cell cycle analysis was conducted by one study that found glyphosate increased the percentage of cells in the S phase and the expression of ERα, VEGFR2, pERK, PI3K(p85), PCNA, and cyclins in HuCCA-1 cells [63]. Two studies looked at apoptosis and necrosis levels: Gasnier et al. found that R450 (a Roundup formulation) activated caspases 3/7 (apoptosis) in HepG2 cells [47], but Mesnage et al. (2013) found that GBH induced adenylate kinase leakage (necrosis) more than apoptosis in HepG2, HEK293, and JEG3 cells, whereas glyphosate increased caspases 3/7 activity [58].

Taken together, in both categories of human cell studies (breast and non-breast), the majority of studies reported a reduction in cell viability upon exposure to glyphosate, GBHs, or AMPA. However, a subset of studies observed an increase in cell viability, indicating a lack of consensus in the current literature. These results are largely consistent with the cytotoxic effects of glyphosate and GBHs which have also been reported in animal-based studies [64,65,66,67]. Notably, GBHs tended to be more toxic, which could be due to adjuvants in the formulation, as Mesnage et al. (2013) found that adjuvants alone were the most cytotoxic, followed by GBHs, with pure glyphosate being the least toxic [58]. Reporting an opposite trend, De Almeida et al. observed that while Wipeout increased HEC1A cell viability, glyphosate reduced it, and Roundup had no effect [45]. The authors suggested that adjuvants or glyphosate impurities in the Wipeout formulation may have caused this proliferation.

Mixed results across studies might be attributable to varying concentrations used. Some studies showed that glyphosate can induce proliferative effects in hormone-dependent breast cancer cell lines [42,43], while others observed cytotoxicity at higher concentrations but proliferation at lower concentrations [44,63]. For example, Sritana et al. found that glyphosate induced proliferation at the lower concentrations tested (10^−13^ to 10^−5^ M) but was cytotoxic at higher concentrations (10^−3^ to 25 × 10^−3^ M) in HuCCA-1 cells [63]. These lower concentrations fall in the range of glyphosate levels that have been detected in the general population (0.16–7.6 µg/L or 0.95–45 nM, as calculated using glyphosate’s molar mass of 169.07 g/mol) and in occupationally exposed subjects (0.26–73.5 µg/L or 1.5–435 nM) [68], suggesting possible biological relevance.

More research is needed to determine the effects of glyphosate on cell proliferation in humans and human cells at these biologically relevant doses and its potential hormetic effect, where the dose–response relationship is characterized by a low-dose stimulation and high-dose inhibition or toxicity, or otherwise nuanced biological effects. Glyphosate has been reported to have a relatively short half-life in the human body, about 5–10 h [69,70]; thus, certain cells, like those in the breast, might be exposed to even lower concentrations than those found in the environment, in food, or even human cells that have direct exposure to the environment, like those lining the respiratory or alimentary tracts. However, since glyphosate, GBHs, and AMPA are commonly found in the human diet [4,5,6,7,8], glyphosate exposure in humans is a near constant. Although glyphosate exposure might induce cell proliferation and, in this way, may be consistent with carcinogenesis, cells in vitro may behave differently than cells in vivo; hence, the translation of in vitro study results to biological effects in humans may be limited.

Glyphosate has been proposed to act through several estrogen-dependent mechanisms. Coppola et al. found that glyphosate increased E2 (17β-estradiol) secretion in MCF-7 and MCF-12A cells and suggested that this could lead to mammary gland angiogenesis through the activation of vascular endothelial growth factor (VEGF) [71]. Whereas Zhang et al. (2020) found that glyphosate did not interfere in the binding between E2 and ERα in MELN cells [46], Thongprakaisang et al. suggested that glyphosate binds to the ligand site of estrogen receptors in T47D and T47D-KBluc cells, acting like a weak xenoestrogen, since its upregulation of ERα and ERβ was inhibited by an ER antagonist [43]. While Mesnage et al. (2017) also found that an ER antagonist blocked glyphosate’s estrogenic effect in T47D-KBluc cells, their molecular dynamics simulations and calculations indicated that glyphosate activates ERα via a ligand-independent mechanism at concentrations higher than typical human glyphosate exposure levels [42]. However, Coppola et al. observed that glyphosate altered ERα and ERβ expression at biologically relevant concentrations in MCF-7 and MCF-12A cells which could impact mammary gland development [41]. Moreover, there was not a consensus regarding the effects of glyphosate and/or GBH exposure on estradiol-mediated transcriptional activity as Gasnier et al. saw a decrease in HepG2 (estrogen receptor-positive liver cancer) cells [47], Zhang et al. (2020) saw no effect in MELN (estrogen receptor-positive breast cancer) cells [46], and two studies saw an increase in T47D-KBluc (estrogen receptor-positive breast cancer) cells [42,43]. A possible explanation for this discrepancy is that T47D-KBluc cells might have greater glyphosate sensitivity than MELN cells [46]. These reported discrepancies could be attributed to the different cell lines used. Glyphosate and estrogen were also found to have synergistic effects on gene expression that could initiate apoptosis in cerebral and myocardial tissues, increase tumor angiogenesis, inactivate tumor suppressor genes, and disrupt immune surveillance [48]. 

Furthermore, glyphosate and/or GBHs were shown to inhibit aromatase activity [46,47,57,59,60,62], but GBHs were more potent since adjuvants could increase glyphosate’s solubilization, stability, penetration, and bioaccumulation [47,57,59,60,62]. Zhang et al. (2020) suggested that glyphosate inhibits aromatase by binding to an allosteric site [46], while Defarge et al. (2018, 2016) proposed that GBH adjuvants might inhibit aromatase through endoplasmic reticulum membrane disruption or through synergistic interactions with heavy metal contaminants in GBHs [60,62]. It has been shown that heavy metals can demonstrate estrogen-like activity, which can further increase breast cancer risk [72]. Due to these varying hypotheses, more research is needed regarding glyphosate’s potential endocrine-disruptive properties and the associated mechanisms.

Glyphosate can also act via estrogen-independent mechanisms. Coppola et al. suggested that glyphosate reduced intracellular ATP levels by increasing mitochondrial membrane permeability, and thus intracellular calcium concentrations, which reduced ATP synthesis [41]. Furthermore, Roundup was found to deregulate ER-independent pathways related to the cell cycle, DNA repair, and metabolism, which could cause hypoxia, higher amounts of ROS, mutation buildup, and cell death [40]. Glyphosate and GBHs were also found to induce genotoxicity in hormone-independent cell lines [45,47], which could be due to the production of toxic ROS as glyphosate and GBH adjuvants are biotransformed [73]. DNA damage has been shown to cause cancer through downstream effects in cellular pathways [31,32,33]. Glyphosate, Roundup, and AMPA might disrupt the immune system by inducing DNA double-strand and single-strand breaks in human peripheral blood mononuclear cells, including lymphocytes, with chronic exposure potentially contributing to lymphoma and leukemia [74,75,76]. In addition, glyphosate could interfere with the interactions between gut bacteria and the immune system [77]. Most of the claims that are used to justify that glyphosate does not affect humans are based on the fact that glyphosate works by disrupting the shikimate pathway, which is only found in bacteria, plants, and certain fungi [3]. However, the human body has about as many bacterial cells as human cells [78], and the gut microbiome has been shown to have effects on many aspects of our health [79]. If glyphosate affects gut bacteria, which use the shikimate pathway to produce tryptophan, phenylalanine, and tyrosine, with the former two being essential amino acids that humans can only get through their gut bacteria or diet [80,81], it logically follows that glyphosate may also have biological effects in humans. 

Other reviews, which have examined the effects of glyphosate, GBHs, and AMPA on outcomes including non-Hodgkin’s lymphoma (NHL) [15,18], epigenetic changes [82,83], endocrine disruption [84,85,86], reproductive system alterations [87], and fertility [88,89], have depicted potential detrimental effects glyphosate and GBHs could have on biological processes. In 2018, a review conducted by the International Agency for Research on Cancer (IARC) [18] concluded that glyphosate is a “probable human carcinogen”, largely based on four studies showing higher frequencies of NHL in occupationally exposed workers [90,91,92,93]. In 2019, Zhang et al. conducted a meta-analysis that included data from the Agricultural Health Study (AHS) cohort along with five case–control studies [15]. Using the highest exposure groups when available in each study, they reported that the overall meta-relative risk (meta-RR) of NHL in GBH-exposed individuals was increased by 41% (meta-RR = 1.41, 95% CI 1.13–1.75). There are limited studies examining the potential relationship between glyphosate and other cancers. In 2017, Andreotti et al. found no associations between glyphosate exposure and cancer risk among mostly male pesticide applicators in the AHS [94]. An older 2005 study based on the same cohort found no relationship between glyphosate and breast cancer risk in farmers’ wives based on questionnaires that assessed the farmers’ and their wives’ pesticide use [95]. However, the Agricultural Health Study analyses did not include biomonitoring to assess glyphosate exposure levels; instead, exposure was based on glyphosate use as reported by questionnaire.

With regards to the analytical procedures used in the cell studies, there were discrepancies in the assays used. The lack of standardization in these assays poses significant challenges to the comparability and validity of the studies. On a similar note, in the human studies reviewed, urinary concentrations of glyphosate and AMPA were measured using different assays with different limits of detection, which could have influenced our interpretation of the results. A standard method of measuring and reporting glyphosate and AMPA concentrations could promote a more accurate assessment of their levels in human subjects and better comparison of the dosage in cell line treatments. Within this topic, human studies were limited, and most studies reviewed used a limited sample size that constricts the generalization of their findings. Therefore, future research needs to utilize more human-based cell models in addition to human cohort and nested case–control studies with long-term follow-up periods that would enable studies to better support their findings [96]. 

## 5. Conclusions

Glyphosate, GBHs, and AMPA are potential environmental toxicants that have been shown to have biological effects on human cell lines and associations with human outcomes. Human-based cell studies generally found that GBHs were more cytotoxic than glyphosate, and some studies reported that glyphosate induced cell proliferation at lower concentrations. Glyphosate has been proposed to act through both estrogen-dependent and estrogen-independent mechanisms as its effects include potential endocrine disruption and genotoxicity. The limited human studies available indicated that glyphosate and AMPA exposure might increase breast cancer risk via cellular and physiological processes that contribute to mammary carcinogenesis. However, more human studies are urgently needed to better understand the impact of glyphosate and AMPA on human health, especially considering their ubiquity.

## Data Availability

No new data were created or analyzed in this study.

## Abbreviations

Gly	glyphosate
GBH	glyphosate-based herbicide
AMPA	aminomethylphosphonic acid
